# Microscopy of the Teeth

**Published:** 1871-10

**Authors:** S. P. Cutler

**Affiliations:** Prof. of Chemistry, Histology, and Microscopy. New Orleans Dental College.


AMERICAN JOURNAL

OF

DENTAL SCIENCE.

Vol. V. THIRD SERIES--OCTOBER, 1871. No. 6.

### ARTICLE I.

### MICROSCOPY OF THE TEETH.

*Fragilitas, Dentium, Senilis and Hyper Cementosis.*

BY S. P. CUTLER, M. D., D. D. S.

Prof. of Chemistry, Histology, and Microscopy. New Orleans Dental College.

There is a stadium prodromirum or stationary stage in animated nature, of longer or shorter duration, when the balance wheels are in perfect equilibrium in healthy individuals, that stage commencing at full maturity and ending at the commencement of decay and decline—or that period of each individual case when the inorganic begins to predominate over the vital or organic forces; a regular down hill grade or decadence commencing at the top of the hill of life and sliding down the inclined plane of old age, the mineral gradually, stealthily and slowly supplanting the vital elements and forces, until finally the vital yield and leave the mineral in full and complete possession of the organism.

During the developmental periods the vital elements and forces are in the ascendency, that is to say, the vital means the organic in contradistinction to the inorganic or mineral.

The one a physiological, the other a mixed or physiological and pathological condition combined.

The bones and teeth can be more readily studied under the microscope, and changes comprehended than any other tissue, the teeth being more readily accessible than the bone for observation; they will be mainly alluded to in this paper.

All observing dentists know from observation that the teeth of young subjects are whiter, softer, more sensitive, more animal matter and less lime in them, less resistant to acid action or decay than those of adults.

Hence there is observable a structural charge taking place in the teeth in youth, in manhood and in old age, or decline steadily moving on, sometimes, perhaps, pausing for a time, or working more slowly, then again starting with renewed energy and continuing unto the end.

In the young the tubuli and pulp canal are larger than in the adult tooth, in the adult larger than insenility.

As these changes go on in the microscopic structure, changes are going on paripassu in the chemico-organic equivalents the lime preponderating more and more as age advances, the specific gravity becoming greater, the color more yellow, the texture harder, more brittle, less sensitive and less vital, more resistant to acid action because less porous or penetrable owing to occlusion, more or less, of tubuli.

In extreme old age the teeth became darker, pulp canal nearly or quite occluded, also tubuli, non-vital, cool, having no vital heat of their own after closure of tubuli and pulp canal, or only in proportion to degree of closure.

The conditions of the teeth spoken of above are infallible indices of the different stages of human life and by the examination of a tooth.

The experienced dentist ought to be able to point out the stage or period of life of the individual the tooth belonged to at the time of removal, and to approximate the age of the individual at least within one decade from the physical, microscopic and chemical characteristics of the tooth, and until the profession has so advanced it cannot claim to be elevated to the highest degree of attainment within their reach.

There might in theory be made some four cardinal epochs or sub-divisions in the life of the octogenarian of twenty years each or two decades.

The first twenty years would include the developmental period. The second twenty years, the comparatively stationary period, when the elements and forces just about balance each other, of decay and reproduction in well developed and protected organisms. In the weak and feeble, perceptible changes may be recognized.

The third twenty years, or the third period includes the fifth and sixth decades or the down hill grade of life, commencing rather slowly and ending more rapidly, the interdental changes marking the beginning and ending of this epoch, being more strongly marked during this period than any other except it be the first.

The fourth and last twenty years finishing or closing out of life; if the limits have been reached the dental changes will be strongly marked by closing up more or less completely the central and tubular structures where there has been only normal use of them, if abnormally exercised they will be closed sooner. The teeth are more subject to local influences than the other osseous structures, from the fact of their cropping out to the surface and subject to local chemical action, attrition and other influences that do not apply to bones in general.

The fundamental changes in the bones are not so apparent and striking as in the teeth, still the same general influences and changes are going on only not quite so energetically marked.

It is a well known fact to all surgeons that injured bones of the young recover much more rapidly than in middle age, and middle age more rapidly than in old age. In fact, in some cases of extreme old age, there seems to be no tendency to resistance, if at all only extremely slow and the process easily arrested at any time during recovery by disturbing influences, such as motion in a case of fracture.

The difference in this respect in different individuals is very great, owing to a variety of causes, such as habits, hereditary tendencies, temperaments, climate and occupation all exerting modifying influences.

Some mature very early, others later, sometimes some commence to decline by old age sooner than others.

Teeth of the first period named, are whiter and softer than the second period, and those of the second period, whiter and more vital than the third, the third more so than the fourth, all things equal. During the second period the canal and tubes close more or less, varying greatly in different subjects, they become slightly yellow also. During the first period and first decade of the second, there are more decays and loss of teeth than in any subsequent periods of equal time.

So long as the teeth remain firm in their sockets and free from tartar, the growth of cementum is not as great as when teeth become loose and diseased in their sockets.

We never find hypertrophy of cement during the developmental stages, when the teeth are sound and firm in their sockets, but frequently find this condition in cases of diseased sockets either without decay, and more frequently towards the end of this period.

In all such cases the effect is superinduced, and is never a natural tendency until near the close of the second period, not then in a majority of cases.

During the third and fourth periods, this tendency is a natural one, more so in the last than during any other period.

As a general thing when thickening of cement commences in normal conditions, a yellowing of cement manifests itself, sometimes preceeding hypertrophy of cement.

The cemental thickening nearly always begins at apex of fang, and travels upwards with generally a regular taper, sometimes ending abruptly, more frequently where there is looseness of teeth and alveolar absorption.

In many cases there is a bulbous enlargement at end of fang extending not far up.

In some instances after this process has made considerable progress, there occurs necrosis just at the end of fang of greater or less extent stopping at the dentine and leaving a sharp point. This never takes places until after death of pulp, and must be regarded as a vital process, the erosion of cement a chemical one, depending always on acid action at point of necrosis, the tooth in fact is dead all except the cement whenever the pulp is destroyed. This condition may happen where there is no hypertrophy of cement.

The cement as well as enamel becomes yellow, as old age advances, the change in dentine being more structural than in either cement or enamel.

The yellowing of teeth never occurs early in life to any great extent, this is most usually the effect of time.

Hypertrophied cement occurs sometimes as a pathological condition before a tooth is six years old.

When hypertrophied cement occurs in young subjects the process is a rapid one much more so than in advancing age.

As the hypertrophy progresses, there must necessarily be a corresponding absorption to make room for the enlarged fang.

All processes of decay and reproduction are more active in youth than in old age, as there is greater activity of forces both chemical and vital in the young than in the aged. In the process of cementosis, there is going on in the alveolus a double process, one of building on to fang, the other removal of alveolar tissue, one a chemical, the other a vital or nutritive process. The cemento alveolar membrane must carry on a double process during this change, one side generating an acid that carries away the socket, while the other side deposits bone material on the fang.

Hyperdentosis externas never can occur, from the fact that the dentine is always covered up, this can only take place on the inner walls which is the order of dental development, and is a continuation of the normal primary process.

The enamel is the only fixed and unchangeable dental tissue, owing to its extreme hardness and mineral preponderance. Even this tissue undergoes changes to a certain extent of nutrition, disintegration and alternate loss of vitality in extreme age, or where connection with dentine is cut off by decay of dentine underneath.

The idea entertained by many that the enamel is dead matter in youth or early life, seems to me not well founded and contrary to physiological laws.

Calculus, such as tartar around teeth and stone in the bladder, is dead matter, and in nowise has any vital connection with, nor belongs to the system, they are deleterious accumulations and disturbing agencies.

After middle life, if not before, a closing up of tubuli and pulp canal by slow degrees commences, and travels on uninteruptedly so long as life lasts, commencing earlier and progressing faster in some than in other cases, indicating premature old age, depending upon mode of living, occupation, constitutional idiosyncrasies and hereditary peculiarities, and usually occur where there have been many teeth removed; excessive use of tobacco may bring on this condition earlier, even in good constitutions.

Precisely how this process of acclusion or secondary growth takes place cannot be so readily accounted for.

Whether or not there is improvised layers of colloid cells which ossify as fast as improvised. As in other bones, this internal change in teeth is not so readily determined and needs farther observation to determine this point. From the slowness of the process the cell idea would seem problematical.

The closing up of the minute bone cells in advanced age depends on the same law, the process being the same as that of the teeth, and unlike any reparatory process of bone after traumatic injury. On the contrary, it is a degenerative process tending to the mineral kingdom.

The closing up of dentinal tubes and bone cells may be regarded as a presage of decay and a precursor of disolution, a yielding up of vital to chemical laws and forces, a broken balance in the wheels of life, a spent spring and falling weight, and man becomes fossil throughout the hard tissues, and death is the result of displacement of life's forces. The above ideas are suggestive of proof.

Knowing those facts we ought at least to use all measures against premature old age or decay in any power. It should be the duty of scientific men to make investigations in this direction and give rules in directing so as to favor early bone development during growth, and retard it afterwards, or during maturity and decline, which is possible and even probable. By following out the plan in relation to diet given by myself in a paper entitled Physiology and Chemistry of Old Age, read before the American Medical Association at New Orleans in May 1869, and published in the New Orleans Medical Jounal for 1870. I think, if commenced in time, much could be accomplished in relation to longevity. This article was copied into the Dental Register, May No., 1870.

These ideas are new, and perhaps new to all and may not meet with much favor at first, like all other new hypotheses. Still the plan proposed can do no harm, if no good, and may be worthy of candid consideration and trial.

I have been making examinations microscopically and otherwise, of teeth and alveolars of persons ranging from sixty to seventy years of age. I find at the apertures a difference in the degree of closure in similar teeth of different individuals where wear and tear has been about equal, which may be accounted for by constitutional tendencies; the temperaments, bodily and mental habits, have great influence on the hard tisues.

Persons of best constitutions as a rule suffer least; the bones being the best guide, as they are influenced less by local causes than the teeth.

In some specimens of very aged persons I notice that the lacunæ become more rounded, shorter in one direction, sometimes larger in the other, some of the lacunæ or bone cells are much smaller than in middle age.

The canaliculi are more indistinct, the anastomosing not well defined, the length not so great, their size smaller and communication from lacunæ to lacunæ to a great extent cut off, and bone nutrition in consequence cut off; also the life of the bone ebbs, the bones become brittle, cool, harder, heavier, less vital, repairs from injuries slower and uncertain. This condition of the bones is felt by the flesh and fluids throughout the entire body. In some bones and regions of the same bone, there may be some slight difference in structural change as in teeth; it is often noticeable in the same person.

In what precise manner these structural changes in the microscopic anatomy are brought about is of difficult solution; the fact of the mineral preponderance is easily determined from data already stated. The histology of these secondary osseous changes are more difficult of comprehension than primary bone histology, more especially in the microscopic structures.

The microscope reveals the structures only, and our conclusions in relation to cause and effect must be based upon comparative analysis, that is, by comparing specimens under the microscope, of bones and teeth of different stages of life and draw our deductions therefrom, which has been given in this paper, so far as my own observations are concerned.

These observations have been made from teeth and bones of persons from six years old to eighty years and upwards, and all intermediate periods.

In some cases the bones and teeth of forty and sixty years show but slight difference, and we naturally conclude that the chances of the individual of sixty years for a given term of years are about equal to the one of forty.

In other words one person at forty years may be as old as the one at sixty, owing to constitutional tendencies.

Jenkins, who died in London many years ago at one hundred and sixty-nine years of age, was vigorous to the end of his life, and at one hundred and fifty years old was as young as ordinary men of good sound constitutions at fifty years, and I have no doubt about that man having good sound teeth up to the last, and if he ever lost any at all it must have been from accident; tartar, even, could have no abiding place in his mouth. His mode of living would be of interest to science.

The above report is a special instead of a general one, though general ideas and facts may be deducted therefrom.

Any special subject relating to anatomy, physiology or histology, or connected with the whole, and any paper of this kind relating to the whole, must, necessarily be long if in any way complete or comprehensive. In consequence I selected a special subject which I think of sufficient import if properly studied. The use of the microscope in scientific organic analysis is at this day too well understood to require comment to any extent. Without the aid of this instrument little could be known of minute structures, or histological knowledge. In fact histology is that branch of physical science which is the result of microscopic revelations, and which as a special branch is of but recent origin, still in its infancy. Anatomy now is divided into Descriptive, Pathological, Surgical and Histological, all intimately blended and connected, and to fully understand any one branch, all the others must be studied and comprehended. I am fully aware, that, as a general thing that even in our colleges, these branches are not studied as they should be in order to send out competent students to practice a special profession, which is intimately connected with more general subjects than is generally taught. Even among the profession of long years of practical experience, how many are there who have taken the pains to fully read upon all those branches, without which knowledge and full competency cannot be claimed. They certainly are not over numerous. These branches only furnish the basis of other studies connected with our specialty.

